# Correction: A multicenter, double-blind, randomized, parallel-group, placebo-controlled study to evaluate the efficacy and safety of camostat mesilate in patients with COVID-19 (CANDLE study)

**DOI:** 10.1186/s12916-022-02695-5

**Published:** 2022-12-08

**Authors:** Taku Kinoshita, Masahiro Shinoda, Yasuhiro Nishizaki, Katsuya Shiraki, Yuji Hirai, Yoshiko Kichikawa, Kenji Tsushima, Masaharu Shinkai, Naoyuki Komura, Kazuo Yoshida, Yasutoshi Kido, Hiroshi Kakeya, Naoto Uemura, Junichi Kadota

**Affiliations:** 1 Department of Pulmonary Medicine, International University of Health and Welfare Narita Hospital, Narita, Japan; 2 Present Address: Respiratory Medicine, Chiba Rosai Hospital, Chiba, Japan; 3 Department of Respiratory Medicine, Tokyo Shinagawa Hospital, Tokyo, Japan; 4 Tokai University Tokyo Hospital, Tokyo, Japan; 5 Department of General and Laboratory Medicine, Mie Prefectural General Medical Center, Yokkaichi, Japan; 6 Department of Infectious Diseases, Tokyo Medical University Hachioji Medical Center, Hachioji, Japan; 7 Department of Respiratory Medicine, Mishuku Hospital, Tokyo, Japan; 8 Clinical Development Planning, Ono Pharmaceutical Co., Ltd., Osaka, Japan; 9 Department of Statistical Analysis, Ono Pharmaceutical Co., Ltd., Osaka, Japan; 10 Department of Parasitology and Research Center for Infectious Disease Sciences, Graduate School of Medicine, Osaka City University, Osaka, Japan; 11 Department of Virology and Parasitology, Graduate School of Medicine, Osaka Metropolitan University, Osaka, Japan; 12 Research Center for Infectious Disease Sciences, Graduate School of Medicine, Osaka Metropolitan University, Osaka, Japan; 13 Department of Infection Control Science, Graduate School of Medicine, Osaka City University, Osaka, Japan; 14 Department of Infection Control Science, Graduate School of Medicine, Osaka Metropolitan University, Osaka, Japan; 15 Department of Clinical Pharmacology and Therapeutics, Faculty of Medicine, Oita University, 1-1 Idaigaoka, Hasama-machi, Yufu-shi, Oita-ken 879-5593 Japan; 16 Department of Respiratory Medicine and Infectious Diseases, Faculty of Medicine, Oita University, Oita, Japan; 17 Nagasaki Harbor Medical Center, Nagasaki, Japan

**Correction: BMC Med 20, 342 (2022)**

**https://doi.org/10.1186/s12916-022-02518-7**

The original article [1] contained a small typo in Masaharu Shinkai’s name which has since been corrected.
